# Cataract surgery reduces intraocular pressure but not posture-induced intraocular pressure changes in patients with angle-closure glaucoma

**DOI:** 10.1038/s41598-019-50598-y

**Published:** 2019-10-01

**Authors:** Pei-Yao Chang, Jia-Kang Wang, Hsin-Yu Weng, Shu-Wen Chang

**Affiliations:** 1 Department of Ophthalmology, Far Eastern Memorial Hospital, Ban-Chiao, New Taipei City, Taiwan; 2 Department of Medicine, National Taiwan University Hospital, Taipei, Taiwan; 3 Department of Healthcare Administration and Department of Nursing, Oriental Institute of Technology, New Taipei City, Taiwan China; 4 Department of Electrical Engineering, Yuan Ze University, Taoyuan City, Taiwan China

**Keywords:** Ocular hypertension, Glaucoma

## Abstract

Cataract surgery leads to a sustained decrease in sitting intraocular pressure (IOP) in patients with angle-closure glaucoma (ACG). The purpose of this study is to evaluate whether cataract surgery can also reduce postural IOP changes. We prospectively examined 106 eyes from 53 patients with narrow angles scheduled for phacoemulsification. IOP was measured in the sitting, supine, and lateral decubitus positions using an ICare rebound tonometer before and 1 week, 1 month, and 3 months postoperatively. The mean baseline IOP in the sitting and lateral decubitus positions was 17.9 ± 4.8 mmHg and 21.43 ± 6.44 mmHg, which significantly reduced to 13.52 ± 3.8 and 17.46 ± 3.62, respectively, 3 month postoperatively (p < 0.001). However, postural IOP change (lateral decubitus minus sitting) at 3 months postoperatively was not significantly different from that at the baseline (3.17 ± 2.63 vs. 3.53 ± 3.38 mmHg, p = 0.85). Postural IOP change was not associated with preoperative sitting IOP, anterior chamber depth, axial length, fixed pupil, or presence of glaucomatous optic neuropathy. Patients with higher preoperative IOP exhibited greater IOP reduction after cataract surgery in every posture (p < 0.0001). In conclusion, cataract surgery reduces IOP in all postures among patients with ACG; however, it does not reduce the magnitude of postural IOP change.

## Introduction

Several studies have demonstrated a modest reduction in intraocular pressure (IOP) following cataract surgery in patients with primary open-angle glaucoma (POAG), ocular hypertension, or primary angle-closure glaucoma (PACG)^1,2^. Recent studies have also suggested that phacoemulsification (PHCE) alone is a feasible option for the long-term control of IOP in patients with ACG^3,4^. However, although we spend one-third of our lifetime sleeping, most studies have focused only on the effect of sitting IOP change after cataract surgery. Therefore, it is important to determine whether cataract surgery also decreases IOP in other non-sitting positions. Moreover, larger posture-dependent IOP changes are related to progression of visual field (VF) defects in glaucoma patients^5^. Therefore, understanding posture-dependent IOP changes can help us to reveal the thorough picture of IOP change and to prevent progressive VF loss caused by occult high IOP during the night time.

Park J.H. *et al*.^6^ reported cataract surgery lowered IOP in the sitting position as well as in the supine and lateral decubitus position in patients without glaucoma. Positional IOP changes in glaucoma patients may not be detected during typical clinic visits. In light of limited data regarding posture-dependent IOP changes after PHCE and intraocular lens (IOL) implantation for ACG, we conducted this study to evaluate whether cataract surgery can also reduce postural IOP changes. Our second aim was to identify possible variables influencing posture-dependent IOP changes. These findings may provide a better understanding of IOP reduction following cataract surgery for PACG.

## Materials and Methods

This prospective study included patients diagnosed with PACG or primary angle closure (PAC). The study was approved by the institutional review board for human research at the Far Eastern Memorial Hospital, New Taipei City, Taiwan (approval no.:104179-E). All methods were performed in accordance with the relevant guidelines and regulations and informed consent has been obtained from every participant. PAC was diagnosed based on an occludable anterior chamber angle with an elevated untreated IOP of >20 mmHg or presence of peripheral anterior synechiae (PAS). PACG was diagnosed based on the same conditions plus the characteristic glaucomatous optic neuropathy. Eyes were excluded in cases with history of trauma, intraocular surgery, or angle closure secondary to other ocular abnormalities. We also excluded patients with 270° PAS up to the Schwalbe’s line, substantial VF defects (mean deviation worse than 20 dB), or acute PAC with edematous cornea and uncontrolled IOP.

All patients received a full ophthalmic examination, including slit-lamp examination, central corneal thickness measurements by ultrasonic pachymetry (SP-100 Handy Pachymeter; Tomey, Nagoya, Japan), ocular biometry measurements by IOL Master V4.8 (Zeiss, Oberkochen, Germany), IOP measurements by Goldmann applanation tonometry (GAT; Haag-Streit AG, Koniz, Switzerland), and perimetry using a Humphrey field analyzer (Humphrey Instruments, San Leandro, CA) according to the central 30–2 program.

A single surgeon (PYC) performed all surgeries using the same surgical technique. Briefly, a 2.2-mm clear corneal incision was made via the temporal approach. After uneventful phacoemulsification and IOL implantation, the cornea incision was closed by hydration. Patients were postoperatively maintained on a tapering dose of topical rinderon for 2 weeks. All anti-glaucoma agents were maintained at the same doses during the study period, except for pilocarpine, which was discontinued 2 weeks before surgery.

Posture-induced IOP changes were measured using an ICare rebound tonometer (ICare PRO; Tiolat Oy, Helsinki, Finland) before surgery and at 1 week, 1 month, and 3 months after surgery. All measurements were performed in the morning. The IOP was first measured in the sitting position (ST IOP); the patient was then instructed to lie on a bed for 5 min and supine IOP was measured. Finally, the patient was instructed to turn to the lateral decubitus position (with the measured eye as the dependent eye). The head was placed on a soft pillow and the body position was maintained for 5 min for IOP measurement in the lateral decubitus position (LD IOP). We measured the LD IOP separately for operated eye and the fellow eye after each side down for 5 mins. Five consecutive sets of measurements with six measurements in each set were acquired. Readings were averaged out to get a single number for each position. The posture-induced IOP change was calculated as LD IOP minus ST IOP.

### Statistical analyses

Paired t-test and McNemar’s test were used to evaluate the differences in IOP between the cataract surgery eye and the contralateral eye. Generalized estimating equations procedures were used to determine if the IOP significantly changed with time during the first three months after surgery. Multivariate regression analysis adjusted for age and sex with postural IOP change or IOP reduction in every posture post-surgery as dependent variables was performed to find possible associated factors. All statistical analyses were performed using SAS (Version 9.4) and SPSS (Version 20) statistical packages. p < 0.05 was considered statistically significant.

## Results

**Figure 1:**
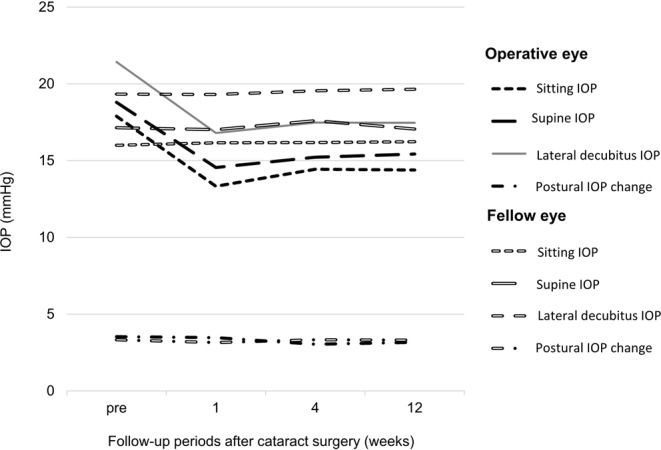
Bilateral IOP change in different body posture before and after cataract surgery.

This prospective study included 106 eyes of 53 patients (mean age, 70.4 ± 7.3 years; 43 females). Baseline characteristics are summarized as means, numbers, and percentages in Table 1. The IOP values of both eyes at each postural position before and after cataract surgery are presented in Fig. 1. Cataract surgery significantly lowered the IOP at one week, and this decrease was maintained until three months post-surgery (Table 2). Alternatively, the postural IOP change did not significantly differ among the various measurement times, including at 3 months post-surgery compared with the pre-surgical baseline IOP(3.17 ± 2.63 vs. 3.53 ± 3.38 mm Hg).

**Table 1:** Baseline characteristics of bilateral eyes before cataract surgery.

	Cataract surgery eye (n = 53)	Fellow eye (n = 53)	*P-value*
OD/OS	29/24	24/29	
PAC:PACG	25:28	35:18	0.13
Central corneal thickness (μm)	551.46 ± 41.71	549.35 ± 38.58	0.58
Anterior chamber depth (mm)	2.46 ± 0.35	2.49 ± 0.31	0.93
Axial length (mm)	22.8 ± 0.65	22.78 ± 0.66	0.46
No. of eyes with attack history	12 (22.6%)	0 (0%)	<0.0001
No. of eyes with fixed pupil	9 (17%)	0 (0%)	<0.0001
No. of eyes with laser iridotomy	32 (60.4%)	29 (54.7%)	0.89
Presence of glaucomatous disc change	28 (52.8%)	18 (33.9%)	0.13
HFA central 30–2 mean deviation (dB)	−13.78 ± −5.65	−12.04 ± −5.11	0.09
Goldmann IOP(mmHg) [range]	17.62 ± 5.72 [9–35]	15.76 ± 3.48 [7–22]	0.02
No. of eyes with glaucoma agents	50 (94.3%)	39 (73.6%)	0.07
Glaucoma agent status			
α2 agonists	20	15	0.62
β blocker	18	11	0.58
Carbonic anhydrase inhibitor	12	9	0.71
Prostaglandin analog	14	8	0.69
Cholinergic	8	0	0.003

McNemar’s test was used in nominal data analysis and paired sample t test was used continuous data analysis.

**Table 2:** Comparisons of IOP change in every posture after cataract surgery.

	Preoperative (n = 56)	1-week (n = 56)	4-week (n = 49)	12-week (n = 41)	*P*-value (pre vs 1- week)	*P*-value (pre vs 12-week)
**Cataract surgery eye**						
Sitting IOP	17.89 ± 4.77	13.33 ± 3.69	14.44 ± 3.29	14.39 ± 3.30	<0.001	<0.001
Supine IOP	18.8 ± 5.11	14.55 ± 3.97	15.22 ± 3.56	15.43 ± 3.35	<0.001	<0.001
Lateral decubitus IOP	21.43 ± 6.44	16.8 ± 3.76	17.48 ± 3.43	17.46 ± 3.62	<0.001	<0.001
Postural IOP change	3.53 ± 3.38	3.47 ± 2.45	3.04 ± 2.89	3.17 ± 2.63	0.85	0.71
**Fellow eye**						
Sitting IOP	16 ± 2.71	16.16 ± 2.83	16.18 ± 2.83	16.23 ± 3.55	0.87	0.89
Supine IOP	17.14 ± 2.96	17.02 ± 3.68	17.6 ± 3.17	17.06 ± 4.08	0.85	0.85
Lateral decubitus IOP	19.33 ± 3.17	19.31 ± 3.52	19.55 ± 3.58	19.65 ± 3.66	0.91	0.79
Postural IOP change	3.33 ± 2.82	3.15 ± 2.39	3.33 ± 2.78	3.31 ± 2.82	0.83	0.89

P-value, paired sample t test.

The IOP of the eye underwent cataract surgery was significantly higher than that of the fellow eye at every posture before surgery, but it significantly lowered after surgery (Table 3). However, posture-dependent IOP changes in the operative eye did not significantly differ from those in the fellow eye either before or after surgery. Multiple regression analysis adjusted for age and sex revealed that posture-dependent IOP changes were not associated with preoperative sitting IOP, anterior chamber depth, axial length, fixed pupil, or presence of glaucomatous optic neuropathy (Table 4). Contrarily, the magnitude of IOP reduction after cataract surgery was related to preoperative IOP, not only in the sitting posture but also in every other tested posture. Thus, patients with higher preoperative IOP tended to exhibit greater IOP reduction after cataract surgery (Table 5). Female patients exhibited significantly greater reduction in sitting (p = 0.02) and supine IOP (p = 0.03) at 3 months post-surgery. The magnitude of IOP reduction was not associated with presence of glaucomatous optic neuropathy or fixed pupil.

**Table 3:** Comparison of IOP in every posture of bilateral eyes before and after cataract surgery.

	Cataract surgery eye	Fellow eye	*P value*
**Preoperative IOP**			
Sitting IOP	17.89 ± 4.77	16 ± 2.71	0.002
Supine IOP	18.8 ± 5.11	17.14 ± 2.96	0.017
Lateral decubitus IOP	21.43 ± 6.44	19.33 ± 3.17	0.036
Postural IOP change	3.53 ± 3.38	3.33 ± 2.82	0.75
**1-week postoperative IOP**			
Sitting IOP	13.33 ± 3.69	16.16 ± 2.83	<0.001
Supine IOP	14.55 ± 3.97	17.02 ± 3.68	0.004
Lateral decubitus IOP	16.8 ± 3.76	19.31 ± 3.52	<0.001
Postural IOP change	3.47 ± 2.45	3.15 ± 2.39	0.33
**4-week postoperative IOP**			
Sitting IOP	14.44 ± 3.29	16.18 ± 2.83	0.001
Supine IOP	15.22 ± 3.56	17.6 ± 3.17	<0.001
Lateral decubitus IOP	17.48 ± 3.43	19.55 ± 3.58	<0.001
Postural IOP change	3.04 ± 2.89	3.33 ± 2.78	0.65
**12-week postoperative IOP**			
Sitting IOP	14.39 ± 3.3	16.23 ± 3.55	0.001
Supine IOP	15.43 ± 3.35	17.06 ± 4.08	<0.001
Lateral decubitus IOP	17.46 ± 3.62	19.65 ± 3.66	<0.001
Postural IOP change	3.17 ± 2.63	3.31 ± 2.82	0.79

P-value, paired sample t test.

**Table 4:** Multivariate regression parameter estimation of postural IOP change.

Postural IOP change	Model 1		Model2	
	Estimate	P-value	Estimate	P-value
Age (per 1 year older)	−0.05	0.42	−0.05	0.46
Female gender	0.34	0.76	−0.20	0.91
Preoperative IOP (per 1 mmHg increase)	−0.07	0.54	−0.09	0.86
Anterior chamber depth (per 1 mm increase)	−1.70	0.17	—	—
Axial length (per 1 mm increase)	—	—	−0.70	0.30
Presence of fixed pupil	−2.06	0.14	−1.41	0.34
Presence of glaucomatous optic neuropathy	0.03	0.97	0.22	0.81

**Table 5:** Multivariate regression parameter estimation of the amount of IOP reduction after cataract surgery.

	Sitting IOP reduction				Supine IOP reduction				Lateral decubitus IOP reduction			
	1 week		3 month		1 week		3 month		1 week		3 month	
	Estimate	P-value	Estimate	P-value	Estimate	P-value	Estimate	P-value	Estimate	P-value	Estimate	P-value
**Model 1**												
Age (per 1 year older)	−0.08	0.28	−0.11	0.11	0.01	0.87	−0.13	0.05	−0.06	0.48	−0.15	0.05
Female gender	−0.33	0.80	−2.34	0.03	−1.08	0.50	−1.98	0.05	−0.55	0.72	−1.73	0.16
Preoperative IOP (per 1 mmHg increase)	−0.70	<0.0001	−0.91	<0.0001	−0.76	<0.0001	−0.68	<0.0001	−1.04	<0.0001	−0.84	<0.0001
Anterior chamber depth (per 1 mm increase)	−0.97	0.49	−0.03	0.98	−0.47	0.78	0.72	0.53	−1.59	0.34	−1.16	0.42
Fixed pupil	1.42	0.36	0.5	0.72	3.18	0.09	2.14	0.09	1.65	0.37	0.88	0.57
Glaucomatous optic neuropathy	0.12	0.91	0.13	0.89	−0.06	0.97	−0.25	0.78	−0.57	0.65	−0.19	0.86
**Model 2**												
Age (per 1 year older)	−0.07	0.30	−0.12	0.09	0.02	0.81	−0.15	0.02	−0.05	0.54	−0.14	0.05
Female gender	−0.38	0.77	−2.48	0.02	−1.08	0.50	−2.1	0.03	−0.63	0.68	−2.13	0.06
Preoperative IOP (per 1 mmHg increase)	−0.70	<0.0001	−0.89	<0.0001	−0.74	<0.0001	−0.70	<0.0001	−1.04	<0.0001	−0.77	<0.0001
Axial length (per 1 mm increase)	−0.35	0.65	0.73	0.25	−0.56	0.55	0.92	0.11	−0.69	0.45	1.74	0.01
Fixed pupil	1.79	0.27	0.31	0.82	3.63	0.06	1.72	0.17	2.33	0.22	0.71	0.63
Glaucomatous optic neuropathy	0.25	0.81	0.36	0.71	−0.06	0.96	−0.07	0.94	−0.36	0.76	0.47	0.64

## Discussion

Previous studies reported that among all body positions, IOP is lowest in the sitting position and highest in the lateral decubitus position^5,7,8^. Further, nocturnal IOP is higher than daytime IOP; this is likely due to the rise associated with the supine position^9^. Piven and Glovinsky^7^ reported that supine IOP was substantially higher than the maximum diurnal sitting IOP. Larger posture-induced IOP changes were also reported to be associated with progression of VF defects^5^. Thus, understanding how posture affects IOP may help develop preventative measures against progressive VF loss caused by untreated or insufficiently treated occult IOP elevation.

Previous studies evaluating postural IOP changes have primarily focused on open-angle glaucoma^5,7,10–12^, whereas few have examined postural IOP changes in PACG patients. Sawada and Yamamoto^13^ compared OAG and PAC eyes and reported no correlation between postural IOP change and axial length. However, contrary to our study cohort, they excluded patients having a history of acute PAC or presence of glaucomatous optic neuropathy. We found the same pattern of posture-induced IOP changes in our investigation; LD IOP was highest in patients with PACG both before and after cataract surgery. Postural IOP changes in our study were approximately 3 to 3.5 mmHg, within the range between 1.6 and 8.6 mm Hg reported in previous studies involving patients with glaucoma^8,14^. Furthermore, we found that neither glaucomatous optic neuropathy nor fixed pupil were significantly associated with posture-induced IOP changes.

Numerous studies have demonstrated sustained IOP reduction after cataract surgery in PACG eyes, ranging from 2.1 to 8.5 mm Hg in chronic PACG^1,3,4^. The mean sitting IOP reduction in this study was approximately 4.1 mm Hg, comparable with the findings of previous studies. These variable IOP reductions among studies may result from differences in patient selection criteria, preoperative IOP, and/or anti-glaucoma drug treatments. We found that patients with higher preoperative sitting IOP exhibited significantly greater IOP reduction following surgery, in accordance with a previous report^4^. We further noted that higher preoperative IOP predicted higher IOP reduction in every position after cataract surgery, not just while sitting. Liu *et al*.^15^ reported deeper preoperative anterior chamber depth (ACD) was associated with higher postoperative IOP in PACG. However, we did not find significant association between preoperative ACD and the amount of IOP reduction for each posture.We further found the amount of IOP reduction for each posture had neither association with axial length, presence of glaucomatous optic neuropathy, or fixed pupil.

Although cataract surgery leads to a sustained IOP decrease in patients with PACG, whether surgery can also reduce diurnal IOP fluctuation remains uncertain. Kim *et al*.^16^ found no diurnal IOP fluctuation change after cataract surgery in patients without glaucoma. However, cataract surgeries were shown to reduce diurnal IOP fluctuations in patients with POAG or pseudo exfoliation with open or occludable angles^17^. Similarly, Tojo *et al*.^18^ reported that cataract surgery decreased IOP fluctuations during the nocturnal period in PACG patients using the contact lens sensor Triggerfish®; however, the mean 24-h IOP fluctuation range did not significantly change after surgery. It is noteworthy, that their study included 10 eyes of which only 6 showed peak values during the nocturnal period. Furthermore, Triggerfish® does not directly provide IOP values in mm Hg; rather, it measures relative changes in mVeq. Park J.H. *et al*.^6^ investigated the effect of PHCE on IOP change in different postures in patients without glaucoma. They reported IOP reduction was 0.6 mmHg, 1.7 mmHg, and 3.0 mmHg in the sitting, supine, and lateral decubitus position, respectively and concluded postoperative IOP reduction was greater in the supine than in the sitting position. (P = 0.048) In their study, postural IOP change (supine IOP-sitting IOP) significantly reduced from 2.0 ± 1.8 to 0.8 ± 2.0 mmHg after PHCE, which differed from our findings that postural IOP change did not decrease after cataract surgery. Several factors may account for this discrepancy. First, our study included only patients with angle closure, which was totally different to their non-glaucoma patients. Cataract surgery has been proved to provide good IOP-lowering effects in eyes with angle closure glaucoma. We did not find greater IOP reduction in recumbent positions was probably because apparent IOP reduction in the sitting position in our study (approximately 3–4 mmHg) comparing to that in their study (approximately 1–2 mmHg). Second, 94.3% of the patients who had PHCE in our study received IOP lowering agents. The effect of these agents on postural IOP change cannot be excluded. Although numerous studies have reported good IOP control and angle widening following lens extraction for PACG, it is still inconclusive whether lens extraction can slow down PACG progression^19^. Large IOP fluctuations were found to be associated with progressive PACG comparing with PACG with stable IOP^20^. Lens extraction did not reduce postural IOP change, thus suggesting that ophthalmologists should carefully monitor glaucoma progression even when sitting IOP was reduced after surgery.

Sawada *et al*.^12^ compared trabeculectomized to conservatively medical treated eyes in patients with sitting IOP < 11 mm Hg and found that the posture-induced IOP change was comparatively smaller in trabeculectomized eyes. Posture-induced IOP change has been attributed to choroidal vascular congestion and increased episcleral venous pressure and might be unrelated to aqueous production^8^. Trabeculectomy creates a new aqueous outflow pathway through the filtering bleb, independent of the episcleral veins; alternatively, lens removal merely widens the angle whereas the drainage pathway is still physiologically unchanged. Lens removal does not alter choroidal vascular congestion or episcleral venous pressure. This may explain our finding that cataract surgery could not reduce posture-induced IOP change. Trabeculectomy was shown to reduce postural IOP change; however, it was not determined whether this decrease was attributable to improved outflow capacity conferred by trabeculectomy or to lower IOP per se^12,14^.

Rebound tonometry is an easy and quick method for measuring IOP in different postures and can be used without topical anesthesia. The probe of the ICare rebound tonometer has a smaller profile than that of a Goldmann prism; hence, it is more suitable for Asian patients with ACG, who usually have deeper-set eyes and smaller palpebral fissures by our own observation. Moreover, rebound tonometry has shown good agreement with dynamic contour tonometry and Goldmann applanation tonometry^21^. Rebound tonometry has been reported to overestimate IOP in cases with higher IOP^22^; however, the mean sitting IOP of the surgical eye in this study was approximately 17 mm Hg, which is in the normal range.

The current study has several limitations. First, IOP spontaneously varies over a 24-h cycle. Although supine IOP was reported to be substantially higher than maximum diurnal sitting IOP^7^; however, our postural IOP change results do not entirely represent the actual nocturnal IOP change because we measured IOP only between 9:00 AM and 12:00 PM during the morning clinic. Second, some patients were taking ocular hypotensive drugs during the study period, and the effects of these agents on postural IOP change cannot be excluded. However, there was no significant relationship between the use of topical hypotensive agents (latanoprost, timolol, and brizolamide) and postural IOP changes in patients with NTG^11^. Third, blood pressure (BP) was not measured, and some studies have found effects of BP on posture-induced IOP changes^23,24^.

Our results indicate that cataract surgery reduces the IOP of patients with PACG in every posture. However, cataract surgery did not reduce the magnitude of the postural IOP change. The magnitude of IOP reduction in every posture after cataract surgery was related to the preoperative IOP, but not to age, sex, ACD, AL, existence of glaucomatous optic neuropathy, or fixed pupil. Our findings provide a more comprehensive evaluation of IOP changes after cataract surgery by including body positions assumed during daily life in addition to the sitting position. These findings provide better understandings of the IOP reduction effect of cataract surgery in patients with PACG.

## Data Availability

The datasets generated during and/or analysed during the current study are available from the corresponding author on reasonable request.
